# Exosomes in Cancer Diagnostics

**DOI:** 10.3390/cancers9010008

**Published:** 2017-01-12

**Authors:** Young Hwa Soung, Shane Ford, Vincent Zhang, Jun Chung

**Affiliations:** Department of Pathology, Stony Brook Medicine, 101 Nicolls Road, Stony Brook, NY 11794, USA; younghwa.song@stonybrookmedicine.edu (Y.H.S.); shane.ford@stonybrook.edu (S.F.); vincent.zhang@stonybrook.edu (V.Z.)

**Keywords:** exosomes, cancer, biomarkers

## Abstract

Exosomes are endosome derived extracellular vesicles of 30–120 nm size ranges. Exosomes have been identified as mediators of cell-to-cell communication by transferring bioactive molecules such as nucleic acids, proteins and lipids into recipient cells. While exosomes are secreted by multiple cell types, cancer derived exosomes not only influence the invasive potentials of proximally located cells, but also affect distantly located tissues. Based on their ability to alter tumor microenvironment by regulating immunity, angiogenesis and metastasis, there has been growing interest in defining the clinical relevance of exosomes in cancers. In particular, exosomes are valuable sources for biomarkers due to selective cargo loading and resemblance to their parental cells. In this review, we summarize the recent findings to utilize exosomes as cancer biomarkers for early detection, diagnosis and therapy selection.

## 1. Introduction

Exosomes are small sized (30–120 nm) extracellular vesicles (EVs) with a multi-vesicular endosomal origin [1,2,3]. Exosomes are distinguished from microvesicles that are heterogeneous in size (50–1500 nm) and shed directly from the budding of the plasma membrane [4,5]. Exosomes play an essential role in cell-to-cell communication by carrying their contents, including proteins, metabolites, RNAs (mRNA, miRNA, long non coding RNA), DNAs (mtDNA, ssDNA, dsDNA) and lipids [6,7]. Intercellular communication mediated by exosomes not only participates in the regulation of normal physiological processes, but also in pathological processes of many diseases including cancer [8,9,10]. They are secreted from most cell types and released in bodily fluids such as urine, plasma, saliva, and breast milk [7,11].

Due to their presence and stability in most bodily fluids and resemblance of their contents to parental cells, exosomes have a great potential to serve as a liquid biopsy tool for various diseases [12,13]. In particular, cancer derived exosomes likely serves as biomarker for early detection of cancer as they carry the cargo reflective of genetic or signaling alterations in cancer cells of origin [14,15,16]. Exosome based liquid biopsy merits consideration over conventional tissue biopsy for following reasons. It provides the convenient and non-invasive way of diagnosis over tissue biopsy that requires surgery. The small sample size of tissue biopsy cannot provide the detailed information of genetic heterogeneity within the primary tumor or metastasized secondary tumors. However, exosomes shed from heterogeneous cancers can be collected at once and provide the dynamic information from the tumors at the time of blood drawing. In this review, we summarize the recent progress of the studies that identify exosome-derived analytes that represent a molecular signature of altered states of cancers for diagnostic purposes. Strategies for isolating exosomes will be presented with an emphasis on their clinical use.

## 2. Isolation of Exosomes

The major hurdle in the clinical utilization of exosomes has been the lack of consistent and dependable methods to isolate a pure exosome population [17]. Therefore, it is essential to overcome technical challenges associates with purifying exosomes to homogeneity free from other subtypes of EVs such as microvesicles and other soluble contaminants to utilize exosomes as cancer biomarkers [18,19,20]. Greeing et al. evaluated and compared the exosome isolation strategies including differential ultracentrifugation (DC), density-gradient separation (DGS), and affinity capture (AC) procedures [21]. The differential ultracentrifugation (DC) has been widely used as conventional isolation techniques that separate exosomes and other EVs based on their sizes and buoyant density. Cell debris and other objects larger than exosomes and shed microvesicles (sMVs) are removed by 1000× *g* centrifugation. Next, over 100,000× *g* ultracentrifugation is used to pellet crude exosomes and sMVs mixtures [22]. The downsides of DC include labor and time consuming procedures, a low recovery yield and low specificity, and no separation of exosomes from microvesicle debris, high molecular weight protein oligomers/protein-RNA complexes, and viruses [17,23]. Density-gradient separation (DGS) fractionates EVs based on buoyant density using a discontinuous gradient of a sucrose solution or Opti-prep. While DGS offers higher purity and recovery rate than DC, it still cannot separate exosomes from sMVs or viruses because their buoyant densities overlap [22].

As an alternative strategy to ultracentrifugation, nanomembrane based ultrafiltration and microfiltration were used to successfully isolate urinary exosomes [24,25]. Recently, exosomes from blood serum were successfully isolated by one-step precipitation method by using commercially available reagents such as ExoQuick (System Biosciences, Palo Alto, CA, USA) as an alternative approach to ultracentrifugation [26]. In this study, Rekker et al. found that the ExoQuick precipitation method not only saves time and labor, but is more efficient than ultracentrifugation in exosomal miRNA profiling [26]. Ongoing efforts that combine Enzyme-linked immunosorbent assay (ELISA), Western blotting analysis, Flow cytometry analysis (FACS) and EV arrays are being made to better track EVs based on their biogenesis mechanism (microvesicles with plasma membrane budding vs. exosomes with endocytic origin) [27,28,29]. These affinity capture (AC) methods target established exosomal markers such as CD63, CD81, TSG 101, HSP 70 and Alix, which allows selective capturing of exosome population followed by detection of cancer specific markers [17].

Affinity capture (AC) approaches rely on exosome surface antigen targeting monoclonal antibody (mAb) that is covalently fused to either magnetic or agarose beads. The multiple reports demonstrated the success of mAbs as baits to isolate exosomes [21,30,31]. In comparison to size-based purification, AC reduced the contamination of cell-debris, protein aggregates and sMVs from exosomes, and allowed to isolate specific sub-population of exosomes by targeting a specific exosomal surface marker [30,32,33]. Tauro et el. performed a proteome study of exosomes isolated from colorectal cancer cell line LIM1863 culture supernatant to evaluate each method mentioned above [22]. They concluded that AC using magnetic beads coated with a mAb targeting exosome surface antigen was far superior to DC and DGS in terms of isolating exosomes [22]. Combination of AC with a microfluidic chip can improve a recovery rate and reduce the sample size to be processed. For example, Chen et al. developed an AC-based microfluidic device that rapidly and selectively capture exosomes from tissue culture and plasma [34]. The AC-based microfluidic device has a great potential for use as a diagnostic tool if it is coupled with downstream analysis techniques to quantify exosomes captured in a device. More examples of microfluidic devices that capture exosomes for diagnostic purposes will be presented in Section 4 and Section 5.

## 3. Detection of Exosomes

Another challenge in exosome biology is how to accurately measure the quantity and purity of exosomes. Exosome purity is assessed by measuring exosome-specific marker antigen or protein (i.e., CD63) as a ratio of exosome concentration (i.e., protein to particle ratio) using ELISA assay [27]. Optical methods such as nanoparticle tracking analysis (NTA), dynamic light scattering (DLS) and flow cytometry (FACS), as well as non-optical methods including resistive pulse sensing (RPS), surface plasmon resonance (SPR) and transmission electron microscopy (TEM) are currently used to measure vesicles/particle numbers [35].

Malvern has developed NTA-based instruments called Nanosight (LM10, LM20, NS200 and NS500) that allow exosomes to be counted and sized by combining light microscopy and the software that tracks Brownian motion of exosomes. The light scattering mode of Nanosight is used to measure size and its fluorescence mode is used to profile labeled markers in exosomes [36,37]. Nanosight is able to measure particles in the size range of 10 nM to 2 μM, and concentration within the range of 10^6^ to 10^9^ particles per mL. NTA method becomes a gold standard to measure the concentration of exosomes and sMVs. The product called Zetasizer Nano ZS (Malvern Instruments Ltd., Malvern, UK) involves DLS technology that determines diameter of EVs including exosomes by measuring the fluctuations from scattering of coherent laser light through suspension of EVs [38]. Nano ZS is specified to measure particles in the size range of 0.3 nM to 1 μM and more accurate to measure vesicles with lower size (<30 nM). The limitation of DLS includes the distortion of accuracy by the presence of large particles, and its incompatibility to molecular labeling. Therefore, it is not suitable for molecular profiling of exosomes. Flow cytometry has been used to resolve individual exosomes and measure multiple surface markers per exosomes [39]. However, due to the smaller sizes of EV’s relative cells, only the particles larger than 300 nm can be resolved.

Regarding to non-optical methods, TEM overcomes the limitation of the conventional light microscope to resolve exosomes as diameter of exosomes is less than optical wavelength. TEM can resolve individual exosomes due to short wavelength of electrons [40]. TEM can serve as a nice complementary technique to the optical methods mentioned to above by providing morphology of exosomes to be resolved. The RPS method allows measuring single nanoparticles as they are driven through a nanopore [41,42]. A transient change in the ionic current flow is generated while the particles flows through the pore and the change in current is proportional to the size of the particle. Maas et al. demonstrated the tunable resistive pulse sensing (tRPS) platform for direct quantification and size characterization of EVs by using the product called qNano system [43]. This method is ideal for direct quantification of exosomes or microvesicles from bodily fluids as isolation or manipulation of EVs is not required. SPR-based nanosensors recently garnered a lot of attention due to their ability to detect small numbers of molecules [44]. Im et al. developed SPR-based exosome sensor called nano-plasmonic exosome (nPLEX) [45]. Each nanohole array of nPLEX is functionalized with antibodies that recognize exosome surface proteins. nPLEX has been used to differentiate ascites samples from ovarian cancer patients from healthy controls as ovarian cancer cell-derived exosomes were identified for their expression of CD24 and EpCAM [45].

## 4. Exosomal Surface Proteins as Cancer Biomarkers

Exosomes contain a variety of proteins that reflect their origin and alteration of the parental cells. Based on endosome-based biogenesis pathway, exosome specific protein markers include endosome associated proteins (e.g., small Rab family GTPases, annexins and flotillin), proteins involved in exosome biogenesis (e.g., Alix, Tsg101 and ESCRT complex), tetraspanins (e.g., CD9, CD37, CD53, CD63, CD81 and CD82), heat shock proteins (Hsp70, Hsp90) and epithelial cell adhesion molecules (EpCam) [46,47,48,49]. These proteins can serve as putative common exosome markers that can be used to isolate exosomes by immune-affinity capture methods or to measure the purity of an exosome population by Western blotting analysis after isolation procedure. Based on exosome isolation techniques mentioned above, the subsequent studies on proteomic analyses of cancer-derived exosomes led to the identification of potential exosomal markers to serve as a liquid biopsy in breast, prostate, pancreatic, ovarian, colorectal cancers and glioblastoma [15,50,51,52,53,54,55,56,57,58,59,60] (Table 1).

A number of exosomal breast cancer markers have been identified in recent years. Rupp et al. characterized CD24 and EpCAM as tumor-derived exosome markers by using immune-affinity isolation techniques involving anti-CD24 and anti-EpCAM magnetic beads [53]. In exosomes-derived from breast cancer patient serum, only CD24 was detectable, but EpCAM was absent due to metalloproteinase-dependent cleavage [53]. The outcome indicated that exosomal CD24 could serve as a circulating breast cancer biomarker. Moon et al. demonstrated that both EDIL3 and fibronectin in circulating EVs (mostly exosome population) can serve as promising biomarkers of early stage breast cancer by using ELISA methods [50,51]. The levels of exosomal EDIL3 and fibronectin from breast cancer patients who underwent surgery were dramatically reduced, suggesting that EDIL3 and fibronectin in circulating EVs can also serve as treatment response markers [50,51].

In addition to breast cancer, exosomal proteins provide the useful source for biomarkers for a number of other cancers. Khan et al. found that Survivin, a member of inhibitor of apoptosis (IAP), was detectable in plasma-derived exosomes from both normal and prostate cancer patients, but the relative amount of exosomal Survivin is significantly higher in plasma of prostate cancer patients [54]. Plasma-derived exosomes were isolated by ultracentrifugation, followed by ELISA and Western blotting analysis to measure the amount of exosomal Survivin in this study. The subsequent study by Khan et al. showed that exosomal Survivin and its alternative splice variants were also elevated in breast cancer plasma [55], suggesting that exosomal Survivin is an important diagnostic markers for a number of cancers. Urinary exosomes, which are readily accessible by non-invasive means, have been utilized to develop biomarkers for prostate cancer and bladder cancer. Protein profiling of urinary exosomes from healthy and bladder cancer patients by label-free liquid chromatography-tandem mass spectrometry (LC-MS/MS) led to identification of eight urinary exosomal proteins as potential biomarkers for bladder cancer [56]. Nilsson et al. demonstrated that PCA3 and TMPRSS2:ERG, two established prostate cancer markers, are also present in urinary exosomes from prostate cancer patients [57]. Glypican-1, the cell surface proteoglycan, was shown to be present in exosomes isolated by FACS from serum of pancreatic patients in both early and late stages, but not in benign pancreatic disease [15]. Exosomes isolated by ultracentrifugation from ovarian cancer patients’ plasma carried TGF-β1 and MAGE 3/6, but not in exosomes from patients with benign tumors [58].

A number of recent studies reported the success of developing the rapid and high-throughput platform for clinical utilization of exosome-based diagnostics without a purification of exosomes. Kanwar et al. [59] developed “ExoChip”, a microfluidic device that captures and stains exosomes with CD63 antibody and a fluorescent dye. This technology allows quantification of exosomes in standard plate reader and profiling of exosomal miRNA analysis [59]. A microfluidic chip labeled with target (CD63, EGFR, EGFRvIII) specific magnetic nanosensor was used in profiling microvesicles for diagnosis of glioblastoma multitome [60]. Yoshioka et al. [61] established the highly rapid and analytical technique called “ExoScreen” that are comprised of CD9 and CD147 antibodies and photosensitizing beads. “ExoScreen” can detect CD9 and CD147 double positive EVs that are enriched in tissue culture media of colorectal cancer cells and serum from colorectal cancer patients [61]. Zhao et al. developed a simpler microfluidic device called “ExoSearch” chip that allows quantitative isolation of exosmes by using immunomagnetic beads. “ExoSearch” chip was used for liquid biopsy of ovarian cancer by measuring three exosomal tumor protein markers such as CA-125, EpCam and CD24 [52]. Consequently, the advancement of immune-capturing systems in microchips led to a highly sensitive and reproducible detection of circulating cancer markers without using a large volume of samples and time consuming isolation processes of exosomes.

## 5. Exosomal Nucleic Acids as Cancer Biomarkers

Initial analyses of nucleic acids from isolated exosomes identified microRNAs (miRs) and mRNAs as the major components of exosomal cargo [62,63]. Subsequent studies revealed that exosomes contain other species of RNAs such as transfer RNAs (tRNAs) and long noncoding RNAs (lnc RNAs) [64,65]. In addition to RNA species, fragments of both single stranded and double stranded DNAs were identified in exosomes [66,67]. Exosomal miRNAs have been under the most attention among exosomal nucleic acids as cancer diagnosis biomarkers due to their stability against RNase-dependent degradation [68,69,70].

Since the discovery of exosomal miRNAs by Valadi et al. in 2007 [62], pioneering studies have been done to characterize exosomal miRNA as diagnostic markers for cancers. Taylor et al. found that eight miRNAs (miR-21, miR-141, miR-200a, miR-200c, miR-200b, miR-203, miR-205 and miR-214), previously known diagnostic markers for ovarian cancer, were also present in circulating tumor exosomes from ovarian cancer patients [68]. Rabinowits et al. [71] performed miRNA-profiling anaylsis on tumor biopsy samples, exosomes isolated from lung cancer patients and control groups. The study demonstrated the similarity of miRNA patterns between exosomes and tumor biopsy samples from lung cancer patients, but these miRNA patterns were significant different from those of exosomes in control group [71]. It suggested that the potentials of circulating exosomal miRNAs as liquid biopsy markers for lung adenocarcinoma [71]. Several miRNAs showed their potentials as diagnostic markers for esophageal squamous cell cancer (ESCC). Tanaka et al. found that the levels of exosomal miR-21 from ESCC patients were significantly higher than those of patients with benign diseases [72]. Exosomal miR-21 levels also correlate with tumor progression and aggressiveness, suggesting that exosomal miR-21 can serve as biomarker as well as therapeutic target [72]. Exosomal miR-1246 was also identified as diagnostic and prognostic markers for ESCC [73]. While exosomal miR-1246 was significantly elevated in serum of ESCC patients, its level is not increased in tumor biopsy samples, suggesting that exosomal miRNA levels may not necessarily reflect the abundance in the cell of origin [73].

During the last three years, more exosomal miRNAs were identified by using a combination of ExoQuick (System Bioscience) precipitation, ultracentrifugation and commercialized Exo-miR kit (Bioo Scientific, Austin, TX, USA) and RNA seq-based miRNA profiling methods in a number of cancer models including breast, colon, prostate, pancreatic cancers and glioblastoma [74,75,76,77,78,79,80]. Table 2 summarizes the candidate exosomal miRNA markers reported up to date for diagnostic tools for these cancers.

Exosomal DNAs merit consideration as a diagnostic tool as well, due to their ability to carry information regarding cancer specific mutations [66,81]. Kahlert et al. identified the large fragments of double stranded genomic DNA (>10 kb) in exosomes from pancreatic cancer cell lines and pancreatic cancer patients. The whole genomic sequencing revealed the mutations in KRAS and p53 in genomic DNA of pancreatic cancer-derived exosomes, indicating the usage of exosomal DNA sequencing to predict the treatment option and therapy resistance [81].

## 6. Conclusions and Future Direction

Cancer-derived exosomes contribute to cancer progression through enhancing intercellular transfer of cargo that contains proteins, lipids and nucleic acids within the tumor microenvironment. The cargo of exosomes reflects the altered state of original cancers, which qualifies exosomes as minimally invasive biomarkers for early detection, diagnosis and prognosis. Compelling recent literature supports that exosome-based diagnostics provide higher sensitivity and specificity over conventional biopsy or liquid biopsy biomarkers due to their stability in biofluids. In addition, exosomal markers are readily available from most biofluids and recent advances in technology in exosome isolation make exosome-based diagnostics cost and labor effective.

The major obstacle to overcome in the field of exosome diagnostics is to develop optimal methods to isolate pure exosome population. Microvesicle themselves can be a useful diagnostic tool to detect cancers, but the comparison studies will only be possible with isolation techniques that separate these two major EVs into pure exosomal vs. microvesicle populations. Another challenge is to understand the mechanisms that regulate the heterogeneity of cancer exosomes, which will affect the contents of cancer-derived exosomal cargo, and therefore influence the reproducibility of diagnostic outcomes. Despite these concerns, future efforts that combine next generation sequencing of exosomal RNAs and DNAs, proteomic analysis of exosomal surface proteins, and immune-affinity capturing techniques will reach to the next level of exosome utilization for cancer diagnosis.

## Figures and Tables

**Table 1:** Exosomal protein biomarkers from body fluids of patients in pre-clinical and clinical studies.

Exosomal Protein	Tumor	Body Fluid	Isolation/Detection Method	Application	Reference
CD24, EpCAM	Breast	Serum, Ascites fluid	Ultracentrifugation and sucrose gradient/MACs using anti-EpCAM beads	Early diagnosis	[53]
EDIL3	Breast	Plasma	ELISA	Diagnosis/monitoring	[50]
Fibronectin	Breast	Plasma	ELISA	Early diagnosis	[51]
Survivin (Survivn 2B)	Breast	Serum	ExoQuick and ELISA	Diagnosis/prognosis	[55]
Survivin	Prostate	Plasma/Serum	Ultracentrifugation (plasma) ExoQuick (Serum)	Early diagnosis	[54]
PCA-3, TMPRSS2:ERG	Prostate	Urine	Filtration and ultracentrifugation	Diagnosis/monitoring	[57]
Glypican-1	Pancreatic	Serum	Ultracentrifugation and FACS	Early diagnosis	[15]
TGF β1, MAGE 3/6	Ovarian	Plasma	Filtration and ultracentrifugation	Prognosis/therapy monitoring	[58]
CD24, EpCAM, CA-125	Ovarian	Plasma	Microfluidic continuous-flow platform (ExoSearch chip)	Diagnosis	[52]
EGFR, EGFRvIII, CD63	Glioblastoma	Serum	Target-specific magnetic nanoparticles (MNPs) and μNMR system	Therapy monitoring and prediction	[60]
CD147, CD9	Colorectal	Serum	ExoScreen using photosensitizer-beads	Diagnosis	[61]

**Table 2:** Exosomal nucleic acid biomarkers in pre-clinical and clinical studies.

Exosomal NA	Tumor	Body Fluid	Isolation/Detection Method	Application	Reference
miR-101, miR-372, miR-373	Breast	Serum	ExoQuick/qRT-PCR	Diagnosis for TNBC	[75]
miR-21, miR-1246	Breast	Plasma	ExoQuick/qRT-PCR	Diagnosis	[76]
miR-21	Esophageal squamous cell carcinoma	Plasma	ExoQuick/qRT-PCR	Early diagnosis and therapy	[72]
miR-21, miR-141, miR-200a, miR-200c, miR-200b, miR-203, miR-205, miR-214	Ovarian	Serum	Magnetic activated cell sorting (MACs) using anti-EpCAM array	Diagnosis and screening of stage	[68]
miR-4772-3p	Colon	Serum	ExoQuick/qRT-PCR	Prognosis for recurrent stage II, III	[76]
let-7a, miR-1229, miR-1246, miR-150, miR-21, miR-223, miR-23a	Colon	Serum	Ultracentrifugation/qRT-PCR	Diagnosis	[77]
miR141, miR-375	Prostate	Serum, plasma	ExoMiR extraction kit (Serum) Qiagen miRNeasy (Plasma)/qRT-PCR	Diagnosis	[78]
miR-17-5p, miR-21	Pancreatic	Serum, urine	Filtration and Ultracentrifugation/qRT-PCR	Diagnosis	[79]
Mutated KRAS DNA	Pancreatic	Serum	Filtration and Ultracentrifugation/PCR and genome sequencing	Diagnosis/prognosis for personalized medicine	[66]
Snc RNA (RNU6-1) miR-320, miR-574-3p	Glioblastoma	Serum	ExoQuick/qRT-PCR-based array	Early diagnosis	[80]
